# Dihydromyricetin protects HUVECs of oxidative damage induced by sodium nitroprusside through activating PI3K/Akt/FoxO3a signalling pathway

**DOI:** 10.1111/jcmm.14406

**Published:** 2019-05-21

**Authors:** Xiaoying Zhang, Lifang Wang, Lizhi Peng, Xiaoying Tian, Xiaoyuan Qiu, Huan Cao, Qiaohong Yang, Rifang Liao, Fengxia Yan

**Affiliations:** ^1^ Department of Pharmacology, School of Medicine Xizang Minzu University Xianyang China; ^2^ School of Medical Science Jinan University Guangzhou China; ^3^ Department of Pharmacy The Seventh Affiliated Hospital of Sun Yat‐Sen University Shenzhen China; ^4^ Department of Pharmacy Sun Yat‐sen Memorial Hospital, Sun Yat‐sen University Guangzhou China

**Keywords:** apoptosis, atherosclerosis, dihydromyricetin, oxidative stress, sodium nitroprusside

## Abstract

The damage of vascular endothelial cells induced by oxidative stress plays an important role in the pathogenesis of atherosclerosis. Dihydromyricetin (DMY) is considered as a natural antioxidant. However, the mechanism of DMY on endothelial cell injury induced by oxidative stress remains unclear. In this study, we found that DMY could reduce the oxidative damage of HUVECs induced by sodium nitroprusside (SNP), HUVECs pre‐treated with DMY suppressed SNP‐induced apoptosis by reduced ROS overproduction of intracellular, decreased MDA level and elevated the superoxide dismutase activity. Meanwhile, we found that DMY could promote the expression of phosphorylated FoxO3a and Akt, and affect the nuclear localization of FoxO3a, when treated with the PI3K inhibitor LY294002, the effect of DMY was blocked. These data suggest that DMY protects HUVECs from oxidative stress by activating PI3K/Akt/FoxO3a signalling pathway. Therefore, DMY may have great therapeutic potential as a new drug for atherosclerosis.

## 1 INTRODUCTION

Atherosclerosis‐related cardiovascular diseases are the predominant cause of disability and death in both developed and developing countries.^1^ However, the mechanism of atherosclerosis is not fully understood. Many studies have shown that endothelial dysfunction, oxidative stress and inflammation may play a vital function in the occurrence and development of atherosclerosis.^2^, ^3^ Among these mechanisms, endothelial cell dysfunction induced by oxidative stress is the key factor affecting the occurrence and development of arteriosclerosis.^4^, ^5^ Oxidative stress can increase the production of ROS, reduce the inherent antioxidant defence system, and ultimately induce cell damage. Apoptosis of human umbilical vein endothelial cells (HUVECs) caused by oxidative stress, including high Nitric oxide (NO) levels in the vein endothelial, is one of the pathological mechanism causing atherosclerosis‐related cardiovascular.^6^, ^7^ In this study, sodium nitroprusside (SNP) was used as a nitric oxide donor to establish a cell model of oxidative stress to HUVECs.

Dihydromyricetin (DMY), as a flavonoid which is isolated from Ampelopsis grossedentata. It has many pharmacological activities, including anti‐inflammatory, antioxidative, lipid and blood glucose‐regulating effects.^8^ DMY can regulate the serum lipid and lipoprotein levels and eliminate oxygen‐free radicals in hyperlipidemic rat model. DMY could decreases the levels of serum triglyceride, low‐density lipoprotein (LDL) cholesterol and total cholesterol content and increases the level of high‐density lipoprotein cholesterol in the high‐fat diet‐fed rats.^9^ In Addition, DMY can reduce oxLDL‐induced cytotoxicity. Williams et al found that DMY prevents plaque formation in the aortas of ApoE knockout mice fed with HFD.^10^ Liu et al found that DMY has cardioprotective effects via the PI3K/Akt signalling pathway.^11^ Luo et al reported that DMY protects HUVECs from oxidative damage by activating Akt.^12^ These results suggested that DMY could reduce atherosclerosis and suppressed the pathologically related metabolic disorders via the PI3K/Akt signalling pathway.

It is known that PI3K/Akt pathway plays an important role to improve cellular proliferation and inhibits apoptosis. As an important downstream target, FoxO3a participates in crucial cellular processes, including oxidative stress, glucose metabolism, inflammation and apoptosis.^13^ Many studies have shown that Akt inhibition can promote FoxO3a‐dependent apoptosis. For example Resveratrol protects PC12 cells from oxidative damage induced by high glucose through activating PI3K/Akt/FoxO3a signalling pathway.^14^ Tsuchiya et al found that FoxO inhibition in endothelial cells to protect mice from atherosclerosis, Knockout of FoxO in mice revealed that increased oxidative stress and accelerates atherosclerosis.^15^, ^16^ The previous studies have shown that DMY play cardioprotective effects via the PI3K/Akt signalling pathway.^11^, ^12^ However, whether DMY can protect HUVECs against oxidative damage via the PI3K/Akt/FoxO3a signalling pathway remains unclear.

In this study, we used sodium nitroprusside (SNP) to create a cell model of oxidative damage to HUVECs. We investigated the protective effects of DMY on the endothelial injury and its mechanism induced by SNP. Our results indicated that DMY reduced SNP‐induced oxidative damage by activating PI3K/Akt/FoxO3a signal transduction.

## 2 MATERIALS AND METHODS

### 2.1 Materials

Dihydromyricetin (DMY) was purchased from Chengdu Kangbang Biotechnology, sodium nitroprusside, MTT, DMSO, DHE and Annexin V‐FITC/PI were purchased from Sigma‐Aldrich; Endothelial cell medium was from ScienCell; Hoechst 33342 were from Beyotime Institute of Biotechnology. PI3K inhibitor LY294002 was from Calbiochem (La Jolla, CA). And the various antibodies used in the experiments were obtained from Cell Signaling Technology.

### 2.2 Cell culture

Human umbilical vein endothelial cells (HUVECs) were purchased from Science Cell Company. HUVECs were maintained in endothelial cell medium (ECM) supplemented with 5% FBS, 1% growth factors, 1% penicillin/streptomycin and incubated at 37°C with 5% CO_2_ humidified atmosphere. HUVECs at passages 3‐5 were used in all experiments. Cells were seeded in six or 96 well plates and grown until confluent and then synchronized by maintaining them in 0.5% serum overnight before treatment.^17^

### 2.3 MTT assay

Cell viability was estimated using a MTT assay as previously described.^18^ Briefly, HUVECs were seeded in 96‐well plates. After appropriate treatment, the culture medium was removed and replaced with 90 mL of fresh DMEM. 10 mL MTT (5 mg/mL) solution was added to each well and the plates were incubated at 37°C for additional 3 hours, then supernatants were discarded and 100 uL DMSO were added. The absorbance of each well solution was measured at 570 nm using a BIO‐RAD680 plate reader.

### 2.4 Hoechst 33342 staining

HUVECs were seeded into 96 well plates (1‐2 × 10^4^ cells/well). After appropriate treatment, these cells were washed with PBS, fixed with 4% formaldehyde for 10 minutes at 4°C and then stained with Hoechst 33342 (10 μg/mL) for 10 minutes at room temperature. After washing with PBS, The apoptotic rate of cells was then observed using a high content screening system as previously described.^19^

### 2.5 Measurement of reactive oxygen species

Intracellular reactive oxygen species (ROS) generation was evaluated using fluorophotometric quantitation.^17^ Briefly, HUVECs were cultured in 24‐well plates until they grown 70% confluence. After treatments with SNP with or without DMY, the cells were incubated with 10 μmol/L DHE for 30 minutes at 37°C. Fluorescence intensity was estimated using inversion fluorescence microscope, and the relative change was processed with the ImageJ analysis program.

## 3 SOD AND MDA

HUVECs were treated with SNP or DMY, then were washed with ice‐cold PBS and centrifuged at 1000 rpm for 5 minutes. The levels of MDA and SOD were measured using respective assay kits.^17^

### 3.1 Caspase 3 activity assay

After treatment, caspase 3 activity was measured by the Caspase‐3/CPP32 Fluorometric Assay Kit.^20^ Briefly, 200 μg of cell lysate were mixed with fluorogenic caspase‐3 substrate and incubated at 37°C for 2 hours. The samples were measured with a fluorometer at an excitation wavelength of 400 nm and an emission wavelength of 505 nm. The fluorescence intensity of each sample was normalized to the protein concentration of sample.

### 3.2 Flow cytometry assay

To quantify the proportion of viable and apoptotic cells in the different treatment groups, HUVECs (5 × 10^5^/well) were seeded into 6‐well plates. After treatment, the cells were harvested, washed twice with ice‐cold PBS, and then incubated 100 μL 1 × Annexin V work solution containing PI for 15 minutes in the dark at room temperature. Thereafter, 400 μL 1 × binding buffer was added and vortex briefly. The number of apoptotic cells was analysed using a flow cytometry as previously described.^12^

### 3.3 Western blotting

Western blotting was performed as previously described.^17^, ^21^ Briefly, treated cells from different experimental conditions were washed three times with ice‐cold PBS and lysed in RIPA buffer. Equal amounts of protein were separated by SDS‐PAGE and transferred to PVDF membranes. Then the membrane was blocked with 5% BSA for 1‐2 hours at room temperature. The primary antibodies against Akt, phospho‐Akt, FoxO3a, phospho‐FoxO3a, Bcl‐2, Bax, cleaved caspase‐3, LaminB1 and β‐actin were used for detecting respective proteins. The membranes were washed three times with TBS‐T and incubated with horseradish peroxidase‐coupled secondary antibody for 1 hours. The protein bands were visualized using ECL kit. The intensity of the bands was quantified using Image J analysis software.

### 3.4 Statistical analysis

Data were obtained from three independent experiments, presented as mean ± SD. Statistical analysis was carried out using a one‐way analysis of variance (ANOVA) and *t* test, and *P* < 0.05 was considered to be statistically significant.

## 4 RESULTS

### 4.1 DMY protected HUVECs against SNP‐induced cell death

**Figure 1:**
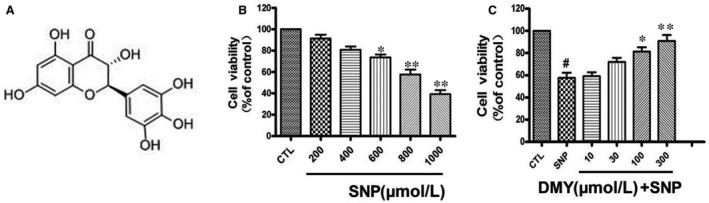
Dihydromyricetin attenuated the decrease in cell viability induced by sodium nitroprusside (SNP) in HUVECs. (A) The structure of Dihydromyricetin. (B) Cells were treated with SNP (200‐1000 μmol/L) for 24 h and cell viability was measured using the MTT assay. (C) Cells were pre‐treated with Dihydromyricetin at indicated concentrations for 2 h and then incubated with or without 800 μmol/L SNP for a further 24 h. Results are shown as the mean ± SD, ^#^ *P* < 0.05 vs CTL group;*P < 0.05, **P < 0.01 vs SNP group

HUVECs were treated with various concentrations of SNP, 24 hours, the cell viability was determined by MTT assay. As shown in Figure 1B, SNP decreased the viability of HUVECs. SNP (800 μmol/L) caused about 40% decrease in cell viability; therefore, this concentration was used in further experiments. To investigate the protective effect of DMY on SNP‐induced HUVEC toxicity, we treated the HUVECs with various concentrations of DMY for 2 hours before exposure to SNP. The result demonstrated that DMY attenuated SNP‐induced cell death. DMY (300 μmol/L) was the most effective concentration (Figure 1C). Thus, DMY (300 μmol/L) was used for the subsequent experiments.

**Figure 2:**
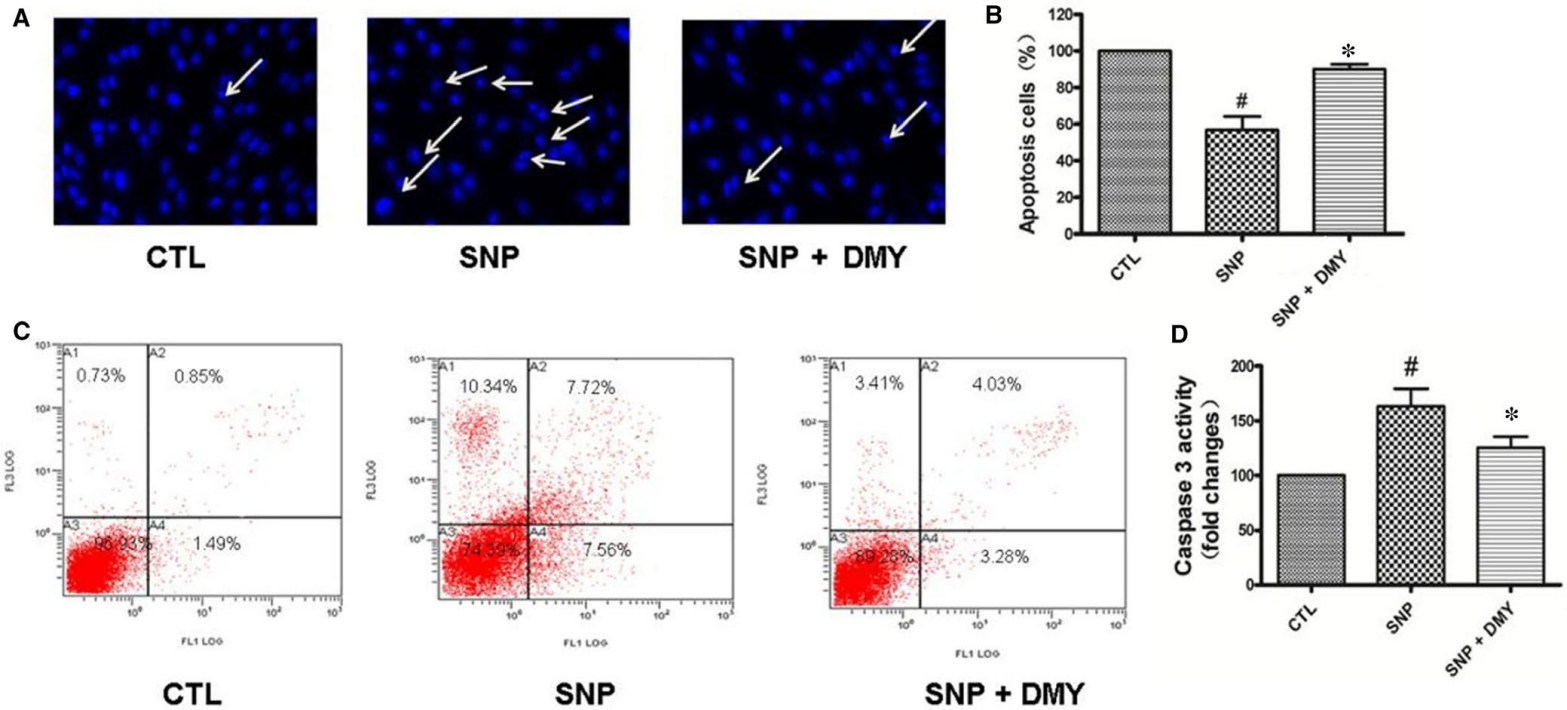
The protective effect of Dihydromyricetin on sodium nitroprusside (SNP)‐induced apoptosis in the HUVECs. Cells were pre‐treated with Dihydromyricetin and then were incubated with or without 800 μmol/L SNP for 24 h. The apoptosis of HUVECs cells was detected by Hoechst staining (A) and Flow cytometry (C), the number of apoptotic nuclei with condensed chromatin was counted from the photomicrographs and presented as a percentage of the total number of nuclei (B); And the activity of Caspase 3 was measured by Caspase‐3/CPP32 Fluorometric Assay Kit (D). Results are shown as the mean ± SD, ^#^ *P* < 0.05 vs CTL group, *P < 0.05 vs SNP group

To verify the cytoprotective effects of DMY on SNP‐induced cell toxicity, HUVECs pre‐treated with DMY were exposed to SNP (800 μmol/L) for 24 hours, then stained with Hoechst 33258. As shown in Figure 2A, the nuclei morphology of the control cells is normal, whereas nuclear chromatin condensation was observed in the SNP‐treated cells, which is an indicator of apoptosis. Statistical analysis showed that DMY (300 μmol/L) pre‐treatment inhibited SNP‐induced nuclear condensation (Figure 2B). To further verify the cytoprotective effects of DMY on the HUVEC apoptosis induced by SNP, Flow cytometry analysis and Caspase‐3 activity were used. As shown in Figure 2C,D, the apoptotic rate of SNP‐treated group was increased substantially (Figure 2C). However, the apoptotic cells were reversed by DMY pre‐treatment. Caspase‐3 activity assay also shown similar results. Therefore, these results suggest that DMY protect HUVECs from SNP‐induced apoptosis.

### 4.2 DMY attenuated SNP‐induced oxidative stress in HUVECs

**Figure 3:**
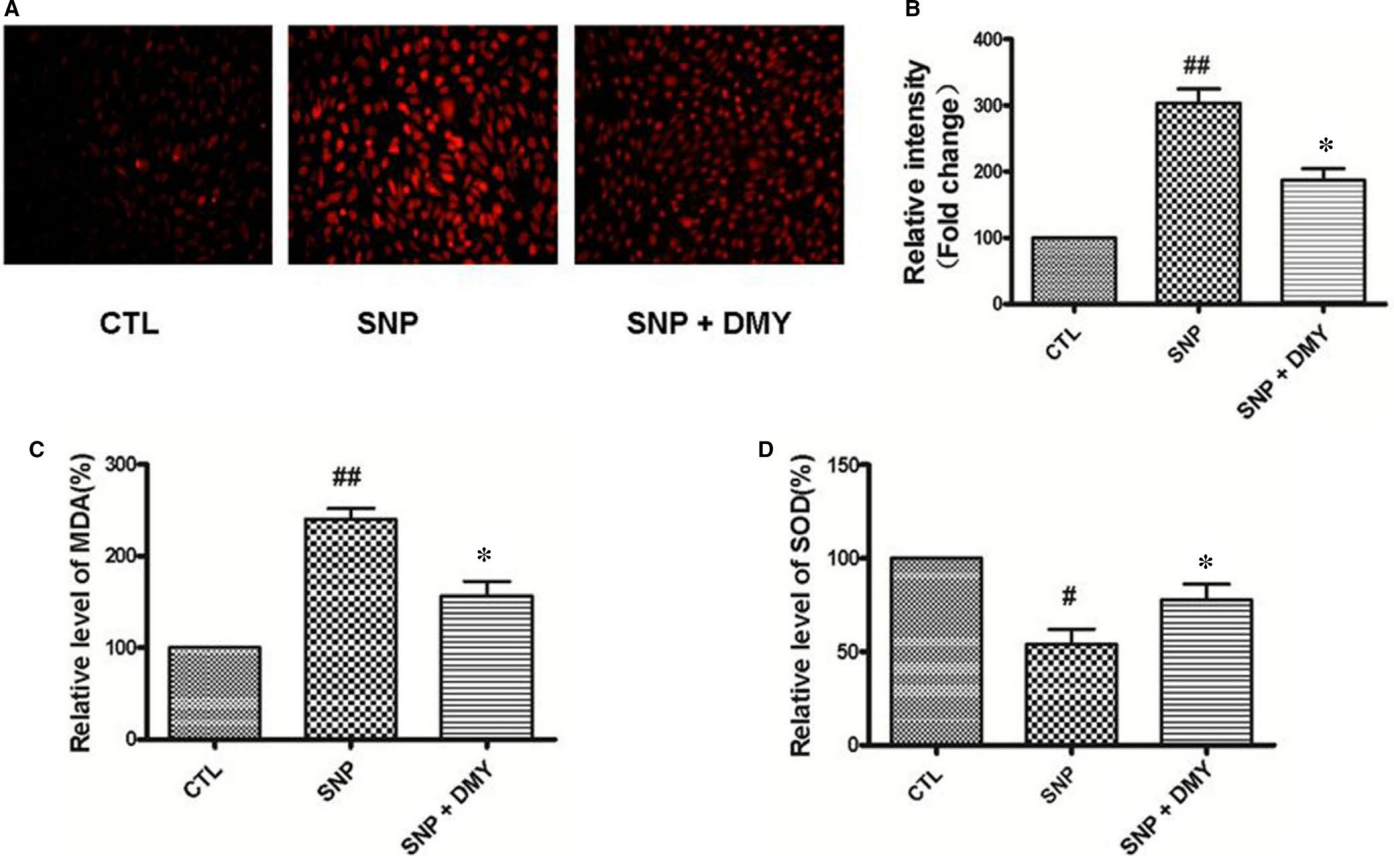
Effect of Dihydromyricetin on sodium nitroprusside (SNP)‐induced oxidative stress in the HUVECs. HUVECs were cultured in 24‐well plates and treated with SNP with or without DMY for 24 h, (A) the cells were incubated with 10 μmol/L dihydroethidium (DHE) for 30 min at 37°C, Fluorescence intensity was detected using inversion fluorescence microscope, (B) and the relative change was processed with the ImageJ analysis. (C, D) The relative level of MDA and SOD was measured by MDA and SOD detection kit respectively. Results are shown as the mean ± SD, ^#^ *P* < 0.05, ^##^ *P* < 0.01 vs CTL group; *P < 0.05 vs SNP group

DCFH‐DA staining was used to detect the ability of DMY to inhibit the generation of ROS. As shown in Figure 3A,B, the HUVECs were pre‐treated with DMY for 2 hours, then exposed to SNP, and the ROS production was decreased. This suggested that DMY reduced the SNP‐stimulated ROS production in HUVECs.

Superoxide dismutase (SOD), as a cellular antioxidant system, can handle the oxidative stress induced by ROS. MDA is used as a biomarker of oxidative stress. To estimate the protective effect of DMY on the SNP‐induce oxidative stress in the HUVECs, the activity of SOD and the contents of MDA were measured with commercial kits. As shown in Figure 3C,D, the MDA level of cells was increased after treated with SNP for 24 hours; and pre‐treated with DMY (300 μmol/L) attenuated this effect. However, the activity of SOD was increased. These results suggest that the protective effect of DMY on SNP‐induce apoptosis is because of the antioxidant of DMY.

### 4.3 DMY protects HUVECs from SNP‐induced apoptosis by activating the PI3K/Akt/FoxO3a pathway

**Figure 4:**
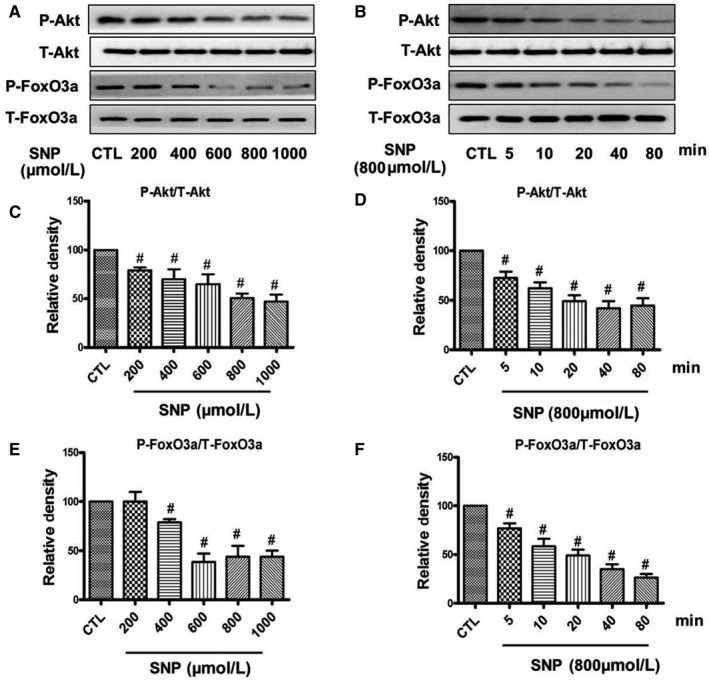
Effects of sodium nitroprusside (SNP) on the phosphorylation of Akt and FoxO3a in HUVECs. HUVECs were treated with different concentrations of SNP for 80 min (A) or treated with 800 µmol/L SNP for various times (B), the phosphorylation of Akt and FoxO3a were analysed by Western blotting. (C‐F) Densitometric analysis of the immunoblot was expressed as a percentage of control. Results are shown as the mean ± SD, ^#^ *P* < 0.05 vs CTL group

As the PI3K/Akt/FoxO3a pathway plays a key role in the stress resistance and apoptosis in many cell types, we investigated the effect of DMY and SNP on the Akt/FoxO3a signalling. First, HUVECs were treated with SNP at different concentrations and different times. The phosphorylation of Akt and FoxO3a was determined by Western blot. The results showed that SNP inhibited the phosphorylation of Akt and FoxO3a, in a time and concentration‐dependent manner in HUVECs. The activation/phosphorylation of Akt and FoxO3a were inhibited in HUVECs by SNP began with 200 μmol/L and reached the maximum effect at 800 μmol/L (Figure 4A). The Figure 4B shows that SNP (800 μmol/L) induced a reduce of the levels of phosphorylated Akt and FoxO3a in 20 minutes, and almost completely eliminates the activation/phosphorylation of Akt/FoxO3a at 80 minutes in HUVECs.

**Figure 5:**
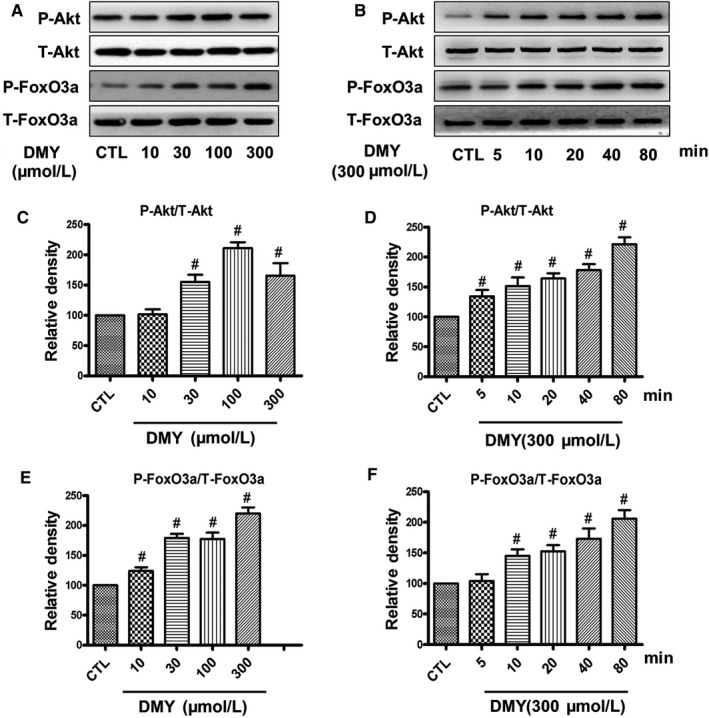
Effects of dihydromyricetin (DMY) on inhibitory effect of sodium nitroprusside (SNP) on the phosphorylation of Akt and FoxO3a in HUVECs. HUVECs were treated with 800 µmol/L SNP with or without DMY for 80 min, (A) phosphorylation of Akt and FoxO3a was analysed by Western blotting. (B, C) Densitometric analysis of the immunoblot was expressed as a percentage of control. Results are shown as the mean ± SD, ^#^ *P < *0.05 vs CTL group; *P < 0.05 vs SNP group

Subsequently, we treated HUVECs with DMY, detected the phosphorylation of Akt and FoxO3a by Western blot. Our results showed that DMY concentration‐ (Figure 5A) and time‐dependently (Figure 5B) augmented the phosphorylated FoxO3a and Akt levels in the HUVECs, the maximum effect was achieved at 80 minutes with 300 μmol/L DMY.

**Figure 6:**
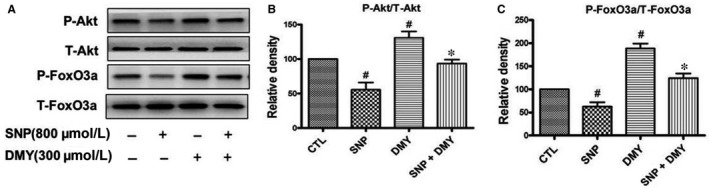
Effects of dihydromyricetin (DMY) on the phosphorylation of Akt and FoxO3a in HUVECs. HUVECs were treated with different concentrations of DMY for 80 min (A) or treated with 300 µmol/L DMY for various times (B), the phosphorylation of Akt and FoxO3a were analysed by Western blotting. (C‐F) Densitometric analysis of the immunoblot was expressed as a percentage of control. Results are shown as the mean ± SD, ^#^ *P* < 0.05 vs CTL group

To study the protective effect of DMY to the SNP‐induced apoptosis in HUVECs, and the effect of the FoxO3a and Akt, we treated cells with DMY (300 μmol/L) before SNP (800 μmol/L), and the phosphorylation of FoxO3a and Akt were determined. Our results showed that the phosphorylation of FoxO3a and Akt was inhibited by SNP in the HUVECs, whereas DMY pre‐treatment could reduce the above inhibition of SNP (Figure 6). It is suggested that the protective effect of DMY on SNP‐induced apoptosis in HUVECs, may be via the PI3K/Akt/FoxO3a pathway.

### 4.4 Effect of DMY on the translocation of FoxO3a and the expression of apoptosis‐related proteins induced by SNP

**Figure 7:**
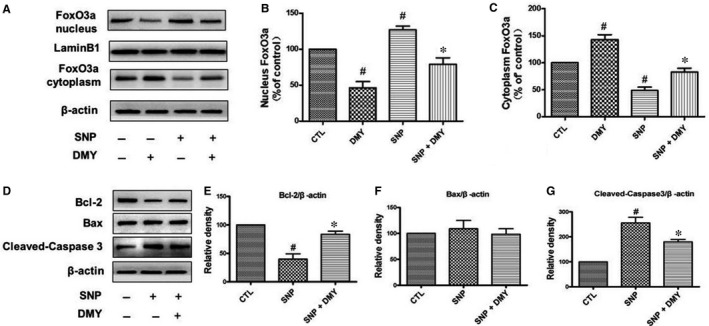
Effects of dihydromyricetin (DMY) and sodium nitroprusside (SNP) in the FoxO3a nuclear translocation and the expression of apoptosis‐related protein. HUVECs were treated with DMY or SNP or SNP + DMY, the nuclear and cytosolic protein of FoxO3a (A) and the level of Bcl‐2, Bax and cleaved‐caspase3 (D) were analysed immunoblotting. (B‐C, E‐G) Relative levels of the nuclear and cytosolic protein of FoxO3a and Bcl‐2, Bax and cleaved‐caspase3 expression in each sample were determined by densitometry of the blots, densitometric analysis of the immunoblot was expressed as a percentage of control. Results are shown as the mean ± SD, ^#^ *P* < 0.05 vs CTL group, *P < 0.05 vs SNP group

Akt is commonly known to phosphorylate/dephosphorylate FoxO3a, which thereafter translocates from cytoplasm to nucleus, where the protein affects the expression of apoptosis‐related proteins and induces cell apoptosis. To explore the effect of DMY on the FoxO3a translocation induced by SNP, we used the Nuclear Extract Kit to extract the cytoplasmic and the nuclear proteins, and the expression of FoxO3a in the cytoplasm and nucleus was measured by Western blot. Results indicated that SNP induced FoxO3a translocation to nucleus from cytoplasm, but DMY reversed the effect of SNP (Figure 7A‐C). To study the protective of DMY on apoptosis‐related proteins induced by SNP, we pre‐treated with DMY in HUVECs before with SNP. The expression of cleaved caspase‐3, Bax and Bcl‐2 was determined. These findings revealed that SNP increased the expression level of cleaved caspase‐3 and dramatically inhibited the Bcl‐2 expression but did not obviously affect the Bax level. However, DMY pre‐treatment effectively repressed these SNP‐induced pro‐apoptotic events (Figure 7D‐G). Therefore, DMY protected the cells from apoptosis induced by SNP through regulating the translocation of FoxO3a and the activity of apoptosis‐related proteins.

### 4.5 LY294002 blocked the protective effect of DMY in SNP‐induced cell apoptosis

**Figure 8:**
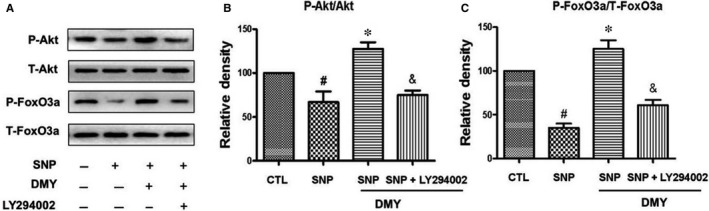
Effects of LY294002 on the phosphorylation of Akt and FoxO3a induced by dihydromyricetin (DMY) and sodium nitroprusside (SNP) in HUVECs. HUVECs pre‐treated with LY294002 were treated with DMY or SNP. The phosphorylation of Akt and FoxO3a was analysed by immunoblotting. (B, C) Relative levels of p‐Akt versus total Akt and p‐FoxO3a versus total FoxO3a in each sample was determined by blot densitometry, densitometric analysis of the immunoblotting was expressed as a percentage of control. Data are shown as the mean ± SD, ^#^ *P* < 0.05 vs CTL group, *P < 0.05 vs SNP group, ^&^ *P* < 0.05 vs SNP + DMY group

To further explore the protective effects of DMY by the PI3K/Akt/FoxO3a pathway on apoptosis induced by SNP, HUVECs were pre‐treated with LY294002, as a PI3K inhibitor, SNP with or without DMY was used before treatment. SNP decreased the phosphorylation of FoxO3a and Akt in HUVECs, whereas DMY reduced the toxicity effect of SNP, the effect of protect was blocked by LY294002 (Figure 8). This result further verified that the effect of protect DMY from apoptosis induced by SNP is mediated by the PI3K/Akt/FoxO3a signal pathway.

## 5 DISCUSSION

Myocardial ischemia caused by atherosclerosis is still the leading cause of death in the world.^22^ Many studies found that oxidative stress‐induced endothelial damage is associated with the initiation and development of atherosclerosis. Oxidative stress increases ROS production, weakens antioxidant systems and ultimately leads to endothelial damage.^6^, ^7^ Therefore, suppressing oxidative stress may effectively inhibit the development of atherosclerosis. DMY is considered as a natural antioxidant, we hypothesize that DMY has a protective effect on the oxidative stress of vascular endothelium. The mechanism of DMY protective on the endothelial cell injury induced by oxidative stress deserves further study.

DMY has a variety of biological activities, including cardioprotective, antioxidant and anti‐inflammatory. The previous studies have shown that DMY has a substantial antihypertensive effect on the pulmonary hypertension.^23^ Many studies have found that DMY could regulated plasma lipid levels and reduced thrombosis. It was also found that DMY could inhibit aldose reductase activity and increased the antioxidant capacity.^24^, ^25^ These studies indicate that DMY has protective effect on oxidative stress injury of vascular endothelium. However, the effect of DMY on SNP‐induced oxidative damage in HUVECs remains unclear. To investigate the protective effects of DMY on the oxidative stress toxicity, we treated HUVECs with SNP to create an oxidative stress model. SNP may because NO overproduction can suppress activity of cytochrome oxidase, leading to the reduction in the electron transport chain and the production of superoxide anions. And excessive oxidative stress damage can lead to apoptosis. That is consistent with previous studies that direct production of nitric oxide (NO) via addition of the NO donor sodium nitroprusside (SNP) to cells caused oxidative stress damage and apoptosis.^26^, ^27^ The results showed that the viability of HUVECs was decreased in a concentration‐dependent manner by SNP (Figure 1B), When DMY was added, the apoptosis of cells were attenuated (Figure 1C). SNP decreased the FoxO3a and Akt phosphorylation, in a concentration and time‐dependent manner (Figure 4). DMY against oxidative stress injury induced by SNP of HUVECs was observed by Flow cytometric analysis, Hoechst 33258 staining and caspase‐3 activity assay (Figure 2A‐D) respectively. It's suggested that the effects of DMY on the oxidative stress toxicity induced by SNP may be correlated with the inhibition of apoptosis.

The previous studies have shown that ROS was related to the pathogenesis of atherosclerosis.^6^, ^28^ The oxidative modification in LDL particles was considered as the essential initial form of atherosclerosis. The lipid oxidation is a ubiquitous process triggered by ROS. Oxidized lipids were associated with biological processes such as inflammation and immunity, and accelerate the pathological process of atherosclerosis. SOD is an important radical superoxide scavenger known to protect cells from oxidative damage.^28^ Meanwhile, MDA is a decomposition product of lipid hydroperoxides, which could be used as an indicator of oxidative damage to cells and tissues.^28^ Some studies have shown that DMY could increase the activities of SOD and decreased the contents of MDA in atherosclerosis animal models and cells.^29^, ^30^ To study the protect effect of DMY in oxidative stress, we investigated the DMY effect of the production of ROS and MDA on HUVECs induced by SNP. According to previous studies, the results showed DMY increased the activities of SOD and reduced the production of ROS and MDA generation in SNP‐induced oxidative damage (Figure 3A‐D). These findings suggested that DMY alleviates SNP‐induced oxidative damage in endothelial cells at least partly by up‐regulating antioxidant enzymes.

**Figure 9:**
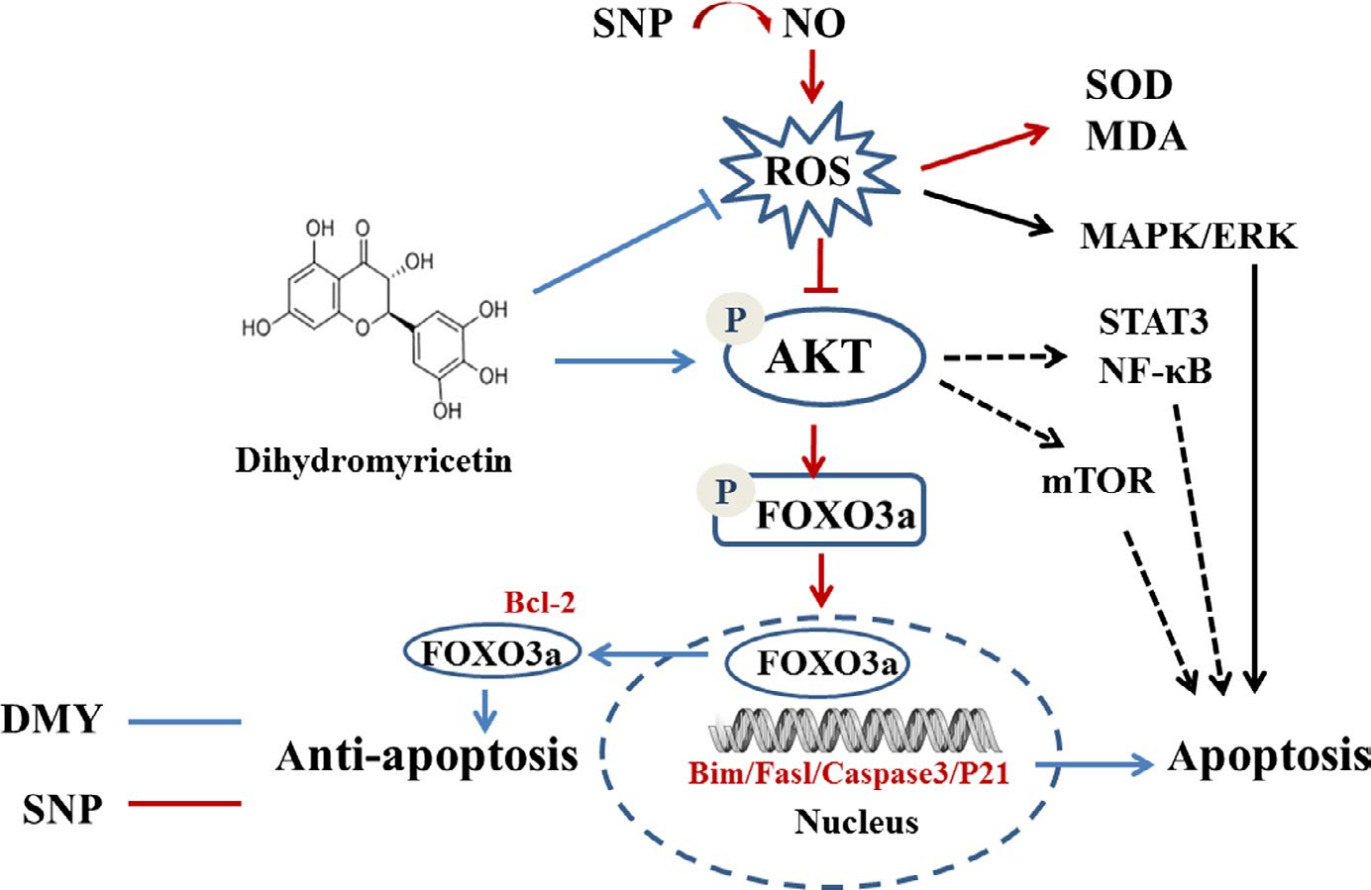
The possibly mechanisms underlying dihydromyricetin (DMY) against sodium nitroprusside (SNP) in the HUVECs. DMY treatment activates PI3K/AKT/ FoxO3a pathway and subsequently drives FoxO3a out of nuclei, which inhibits apoptosis. SNP, a nitric oxide donor, creates oxidase stress and subsequently down‐regulates PI3K/AKT/FoxO3a pathway. Additionally, DMY may modulate others signal pathway such as mTOR, STAT3/NF‐κB to antiapoptosis in the HUVECs

The mechanism of DMY in the treatment of atherosclerosis remains unclear. Which needs to be further explored. Qin's research showed that DMY attenuated atherosclerosis through the Nrf2 signalling pathway and induced HUVEC apoptosis.^31^ Luo's found that DMY protects HUVECs from ox‐LDL‐induced oxidative injury by activating Nrf2/HO‐1 pathway by up‐regulating Akt and ERK1/2.^12^ Zeng's study showed that DMY reduce foam cell formation via activation of LXRα‐ABCA1/ABCG1 signalling pathway.^32^ Here, we focus on the PI3k/Akt/FoxO3a pathway, which plays crucial role in promoting survival and function of cardiomyocyte. The previous studies have shown that hydrogen peroxide induces cardiomyocyte apoptosis by inhibiting the PI3K/Akt pathway.^33^ The PI3K/Akt pathway has shown related to the human atherosclerosis.^34^ FoxO3a is an important downstream target of the PI3K/Akt pathway, which was mainly regulated by Akt. In the presence of serum and growth factors, Akt was phosphorylated and in turn phosphorylates FoxO3a, then FoxO3a was transported from the nucleus to the cytoplasm, where FoxO3a down‐regulates the expression of pro‐apoptosis molecules, such as Bax and Bcl‐2.^35^, ^36^ On the contrary, oxidative stress can induce FoxO3a from the cytoplasm to the nucleus, activate the target genes of FoxO3a, such as the pro‐apoptotic gene Bim, and induce cell apoptosis.^37^, ^38^ The role of FoxO3a in oxidative stress‐induced cardiotoxicity has received attention. For example in HUVECs, high glucose decreases the phosphorylation of Akt and FoxO3a, and induces FoxO3a activation, causes ROS production and cell apoptosis.^39^ To investigate the role of the PI3K/Akt/FoxO3a pathway and its target gene in the protective actions of DMY, we examined the phosphorylation of Akt and FoxO3a and the expression of Bax, Bcl‐2 and cleaved caspase‐3 in HUVECs. The results showed that SNP inhibited the basal levels of phosphorylated Akt and FoxO3a (Figure 4), DMY concentration and time‐dependently increased the phosphorylated Akt and FoxO3a levels (Figure 5). When pre‐treated with DMY (300 μmol/L) with SNP (800 μmol/L), DMY reversed the effect of SNP (Figure 6). SNP induced FoxO3a translocation into the nucleus, increased the expression and activation of cleaved caspase‐3, and inhibited the expression of Bcl‐2, but had no effect on the expression of Bax (Figure 7). When pre‐treated with DMY, these effects were prevented. Furthermore, the effects of DMY were blocked by LY294002 (Figure 8). It's suggested that PI3K/Akt/FoxO3a and its target gene Bcl‐2 and cleaved caspase‐3 may play an important role in the protective effect of DMY against the apoptosis of HUVECs induced by SNP (Figure 9).

In conclusion, our results showed that DMY could reduce the oxidative damage induced by SNP. Furthermore, we found that the PI3K/Akt/FoxO3a pathway was participation in the protective process. As far as we know, the study first demonstrated that DMY could enhance endothelial cell survival induced by SNP through the PI3K/Akt/FoxO3a pathway. Therefore, it is suggested that the PI3K/Akt/FoxO3a pathway may be a therapeutic target in atherosclerosis, and DMY may have a great prospects for treat atherosclerosis.

## CONFLICT OF INTEREST

The authors declare no conflict of interest.
